# Labrador Sea freshening at 8.5 ka BP caused by Hudson Bay Ice Saddle collapse

**DOI:** 10.1038/s41467-019-08408-6

**Published:** 2019-02-04

**Authors:** Annalena Antonia Lochte, Janne Repschläger, Markus Kienast, Dieter Garbe-Schönberg, Nils Andersen, Christian Hamann, Ralph Schneider

**Affiliations:** 1 Institute of Geosciences, Kiel University, Ludewig-Meyn-Straße 10, 24118 Kiel, Germany; 2 GEOMAR Helmholtz Centre for Ocean Research Kiel, Wischhofstraße 1-3, 24148 Kiel, Germany; 3 Max Planck Institute for Chemistry, Hahn-Meitner-Weg 1, 55128 Mainz, Germany; 4 Department of Oceanography, Dalhousie University, 1355 Oxford Street, Halifax, B3H 4R2 NS Canada; 5 Leibniz Laboratory for Radiometric Dating and Stable Isotope Research, Kiel University, Max- Eyth-Str. 11–13, 24118 Kiel, Germany

## Abstract

A significant reduction in the Atlantic Meridional Overturning Circulation and rapid northern Hemisphere cooling 8200 years ago have been linked to the final melting of the Laurentide Ice Sheet. Although many studies associated this cold event with the drainage of Lake Agassiz-Ojibway, recent model simulations have shown that the Hudson Bay Ice Saddle collapse would have had much larger effects on the Atlantic Meridional Overturning Circulation than the lake outburst itself. Based on a combination of Mg/Ca and oxygen isotope ratios of benthic foraminifera, this study presents the first direct evidence of a major Labrador shelfwater freshening at 8.5 ka BP, which we associate with the Hudson Bay Ice Saddle collapse. The freshening is preceded by a subsurface warming of the western Labrador Sea, which we link to the strengthening of the West Greenland Current that could concurrently have accelerated the ice saddle collapse in Hudson Bay.

## Abstract (web summary)

The exact freshwater scenario that caused the 8.2 ka cold event is still debated. This study presents new evidence for a severe Labrador shelfwater freshening 8500 years ago that was caused by the Hudson Bay Ice Saddle collapse following a warming in subsurface waters.

## Introduction

In order to improve future climate models and assess the potential impacts of modern Greenland Ice Sheet melt, it is crucial to obtain a better understanding of past meltwater-induced changes in ocean overturning circulation. A rapid northern hemisphere temperature drop of 1–3 °C^1–6^ 8200 years before present (BP) has been attributed to a major disruption in the Atlantic Meridional Overturning Circulation (AMOC)^7,8^. The disturbance of North Atlantic Deep Water formation, in turn, has been linked to a severe freshening caused by the sudden outburst of ice-dammed proglacial Lake Agassiz-Ojibway^9^ (LAO), which formed along the southern margin of the Laurentide Ice Sheet (LIS) during the terminal stage of the last deglaciation over North America. Erosive channels in the southwestern Hudson Bay seabed suggest that LAO drained catastrophically and subglacially underneath a grounded ice dam^10,11^, dispersing a red-colored, hematite-rich sediment layer^12,13^ from the western to eastern reaches of Hudson Strait^9,14–17^. An estimated 163,000 km^3^ of freshwater^14,18^ would have drained into the North Atlantic in one or two successive pulses^11,14,18^ between 8740 and 8160 years BP^9^. However, many model simulations using a short-lived drainage pulse of LAO volume as a trigger alone^19–21^ fail to reproduce a climate perturbation that lasts 160–400 years, such as seen in many climate proxy records revealing the 8.2 event^3,7,22–24^. In contrast, model studies that have applied higher freshwater volumes and proposing that the initial flood event was followed by longer routing or rapid ice sheet melting were able to reproduce a climate perturbation resembling the 8.2 kilo annum (ka) event^19,25–28^. Moreover, energy mass balance, ice sheet, and climate modeling studies suggest that the collapse of the Hudson Bay Ice Saddle^29^ would have had much larger effects on AMOC strength than the lake outburst itself^30,31^.

**Fig. 1:**
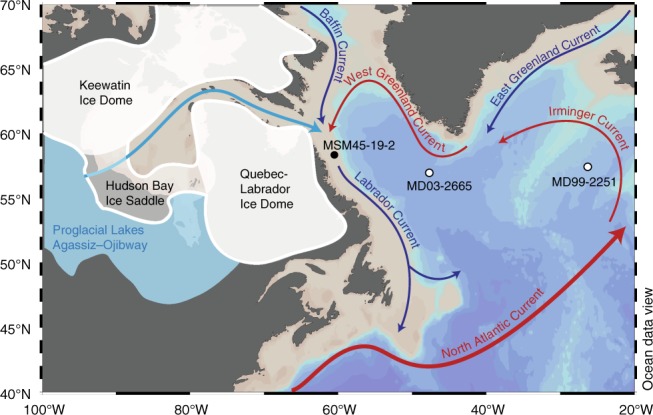
North Atlantic Ocean circulation and sediment core locations. The white shaded area shows the extent of the Laurentide Ice Sheet (LIS) at 8.5 ka before present, with the Hudson Bay Ice Saddle connecting the Keewatin and Quebec-Labrador Ice Domes^32–34^. The blue shaded area displays Lake Agassiz-Ojibway that formed along the southern margin of the LIS and drained through subglacial channels in the Hudson Bay seabed (light blue arrow). Dark blue and red arrows represent cold and warm currents of the North Atlantic, respectively. The Labrador Current originates from a mixture of the cold, fresh Baffin Current and the warmer, more saline West Greenland Current, which is composed of Atlantic waters from the Irminger Current and the cold, fresh East Greenland Current. Core locations are marked with black (this study) and white (other studies) circles. Bathymetric basemap made using Ocean Data View^60^

The Hudson Bay Ice Saddle connected the Keewatin and Quebec-Labrador ice domes of the LIS over southern Hudson Bay (Fig. 1) and was fronted by the largest ice stream of the LIS, the Hudson Strait Ice Stream^32^. In the early Holocene, when the Hudson Strait Ice Stream retreated, Hudson Strait and large parts of northeastern Hudson Bay became ice-free, while a remnant of the Hudson Bay Ice Saddle still formed an ice dam along the continental margin and the marine basin of southwestern Hudson Bay, where it blocked LAO^33^. Though major ice domes remained on land until about 6.7 ka BP^34^, the Hudson Bay Ice Saddle disappeared between 8.5 and 8.4 ka BP^33^, which—according to model studies—would have released freshwater for several centuries^26,35^.

Although direct evidence of the Hudson Bay Ice Saddle collapse is still elusive, a prominent freshwater signal along the proposed drainage route was previously attributed to the LAO drainage event. A sediment core from the central Labrador Shelf recorded a surface water freshening around 8.2 ka BP based on a reduction in the Mg/Ca temperature-adjusted planktic foraminiferal δ^18^O signature^36^. A negative excursion in δ^18^O values of calcitic benthic foraminifera has been detected in a northern Labrador Shelf core also around 8.2 ka BP^37^. A series of older early Holocene freshwater events was described in a central Labrador Shelf core based on peaks in detrital carbonate correlating with reductions in calcitic δ^18^O of planktic and benthic foraminifera^38^. In contrast to these findings, some sediment records along the proposed drainage route did not contain any indication of a prominent freshwater pulse^39^. Such inconsistent evidence combined with limited sampling and dating resolution of previous records have left the exact timing and origin of the most significant freshwater injection ambiguous.

Here, we present a high-resolution and well-dated sediment record from the northern Labrador Shelf (Fig. 1) that provides new evidence of the freshwater signature and the mechanisms involved in the Hudson Bay Ice Saddle collapse. The core site is located in Saglek Basin proximal to Hudson Strait in the proposed drainage route, which under modern conditions is influenced by the inner branch of the Labrador Current that flows along the Labrador Shelf and enters the North Atlantic at Flemish Cap (Fig. 1). Elemental composition and color reflectance data reveal sediment dispersal characteristic of the subglacial drainage of LAO, while a combination of Mg/Ca ratios and stable oxygen isotopes of benthic foraminifera indicate temperature changes and freshening of Labrador Shelf bottom waters.

## Results

### Detecting the freshwater signature

**Fig. 2:**
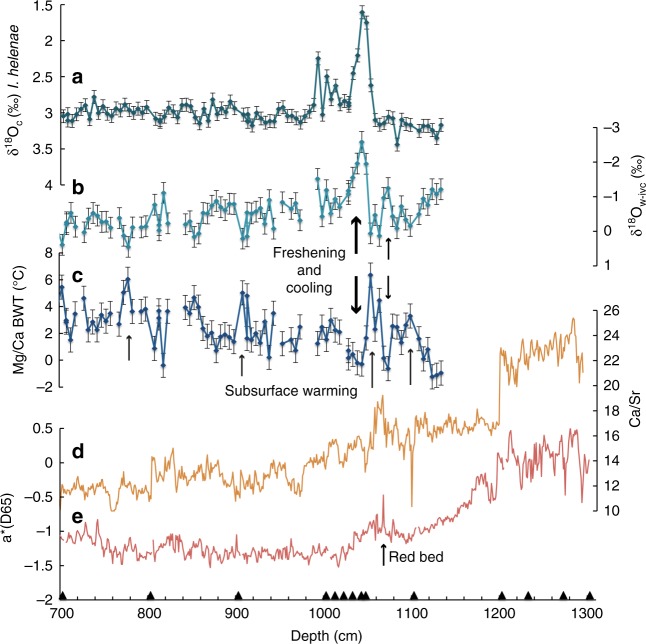
Depth profiles of multiple proxies in core MSM45-19-2. **a** Stable oxygen isotopes, δ^18^O_c_, of benthic foraminifera *I. helenae* displayed with ±0.09‰ uncertainty. **b** Temperature and ice-volume corrected seawater δ^18^O_w-ivc_ displayed with ±0.31‰ uncertainty. **c** Mg/Ca bottom water temperatures displayed with ±0.9 °C uncertainty. **d** Ca/Sr ratios reflect influx of detrital carbonate. **e** a*(D65) indicates red sediment color. Black triangles mark the accelerator mass spectrometric radiocarbon dates

Sedimentological changes associated with the LAO drainage event are determined by characteristic peaks in both redness of bulk sediment (color reflectance a*D65 values) and relative content of detrital carbonate inferred from elemental Ca/Sr ratios measured by X-ray fluorescence (XRF) core scanning (see Methods). The most pronounced peak in Ca/Sr occurs between 1073 and 1068 cm (Fig. 2d), supported by an increase in color reflectance redness (Fig. 2e). The freshwater signal itself is based on a combination of stable oxygen isotopes and Mg/Ca temperature estimates of the benthic foraminifera species *Islandiella helenae* (see Methods), representing subsurface/bottom water conditions at about 200 m water depth. The δ^18^Ο_c_ record is relatively steady, centered at 3.2 ± 0.3‰, with the exception of a large reduction to 1.6‰ at 1048–1043 cm depth and another decrease to 2.25‰ at 1013–993 cm depth (Fig. 2a). The Mg/Ca temperature estimates fluctuate between −1 and 6 °C and show warm peaks at 1100, 1050, 910, and 775 cm depth. The gradual warming from −1 to 3 °C between 1130 and 1100 cm depth and the warm peak to 6 °C at 1050 cm depth precede two cold spells to 0 °C, the first at about 1070 cm depth and the second cooling at 1040 cm depth (Fig. 2c). These two cold spells are supported by two freshenings evident in the temperature and ice volume corrected δ^18^O_w-ivc_ record (Fig. 2b). The δ^18^O_w-ivc_ record ranges from −2.5 to 0.5‰ and shows a minor minimum between 1073 and 1068 cm and a distinct minimum at 1048–1028 cm depth, corresponding to the subsurface water cold events, while the δ^18^O_c_ reduction at 993 cm depth (Fig. 2a) is only reflected by a minor reduction in the seawater δ^8^O_w-ivc_ record (Fig. 2b).

### Chronology

**Fig. 3:**
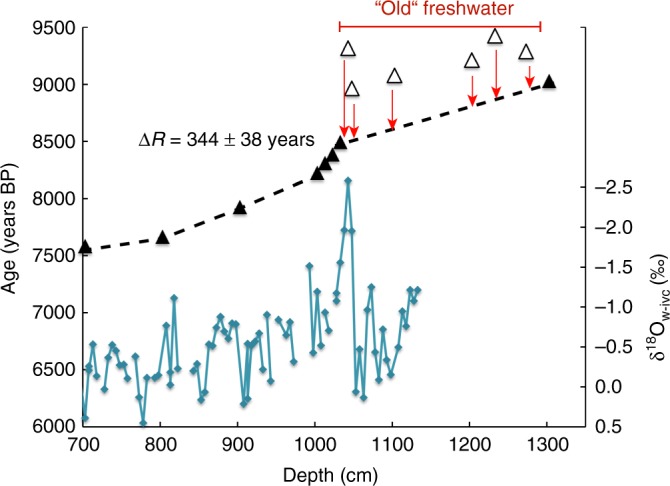
Age–depth relationship of calibrated accelerator mass spectrometric (AMS) dates in core MSM45-19-2. The δ^18^O_w-ivc_ record (blue line) is plotted against core depth (700–1303 cm). All AMS dates were calibrated with Δ*R* = 344 ± 38 years^40,41^. Black triangles mark the calibrated AMS dates that were used for establishing the age model by applying linear interpolation between age tie points. White triangles mark the AMS dates that would fit to this age model only when assuming much higher reservoir corrections in the order of Δ*R* = 600–900 years. Such high reservoir ages could be explained by the dilution of Labrador Shelf waters with Hudson Bay freshwater run-off that contains ancient carbon dioxide from glacier melt or old carbon dissolved from the Paleozoic carbonates that underlie the region^12^

Reservoir corrections are crucial for age calibration. However, quantification of the local reservoir effect is made difficult by its dependence on variable water mass ventilation and—in the case of high latitude records such as the one presented here—on meltwater run-off or seasonal sea ice cover. As the Holocene Labrador Current was prone to large variability due to meltwater run-off from the decaying ice sheet and reorganizations of Labrador Sea circulation^38^, short-lived changes in the local reservoir effect are likely. Based on the present Labrador Shelf marine reservoir correction of Δ*R* = 144 ± 38 years^40^ and an additional early Holocene sea-ice correction of 200 years^17,41^, we applied a Δ*R* of 344 ± 38 years to all accelerator mass spectrometric (AMS) dates below 700 cm depth (Fig. 3). However, six AMS dates between 1043 and 1273 cm depth appear significantly older than the dates at 1033 and 1303 cm depth, making linear interpolation challenging. Additionally, the large age offset between the AMS dates at 1033 and 1043 cm depth would imply an 800-year-long meltwater pulse with low sedimentation rates throughout this interval, which is deemed unrealistic. We rule out that such a large age offset could be the result of reworked material transported to our core site with strong currents or a major meltwater pulse from Hudson Strait, as there are no shifts in elemental composition or grain size (unpublished) to indicate a turbidite, reworked material or any other sedimentological disturbance corresponding to the prominent δ^18^O minimum. Aligning the intermediate dates with the radiocarbon dates at 1033 and 1303 cm depth would require reservoir corrections in the order of Δ*R* = 586–934 years (Fig. 3). Corresponding reservoir ages up to 1,200 years, however, have only been found at present-day high latitudes in the Southern Ocean and the North Pacific but are not substantiated by any published work from the polar or subpolar North Atlantic^40–43^. As the δ^18^O minimum carries the oldest radiocarbon age, followed by a gradual decline in both the stable oxygen isotope record and the radiocarbon ages of the AMS dates, we suggest that the aging effect is related to glacial meltwater increasing the local reservoir effect by diluting Labrador Shelf waters with meltwater from glacial ice that contains no more radiocarbon, since ancient carbon dioxide is stored in glacial ice for thousands of years. Additionally, water masses from Hudson Bay and Strait can contain an older radiocarbon signature due to the dilution with carbon free of ^14^C dissolved from the Paleozoic carbonates that form the bedrock of large parts of the region^12^. Thus, we posit that the interval between 1043 and 1273 cm depth was influenced substantially by glacial meltwater run-off, which would have increased the local reservoir effect. However, we have no other tools to verify such high reservoir corrections at the moment and we therefore choose to disregard these dates for linear interpolation (Fig. 3).

### Pronounced Labrador Shelf subsurface freshening at 8.5 ka BP

**Fig. 4:**
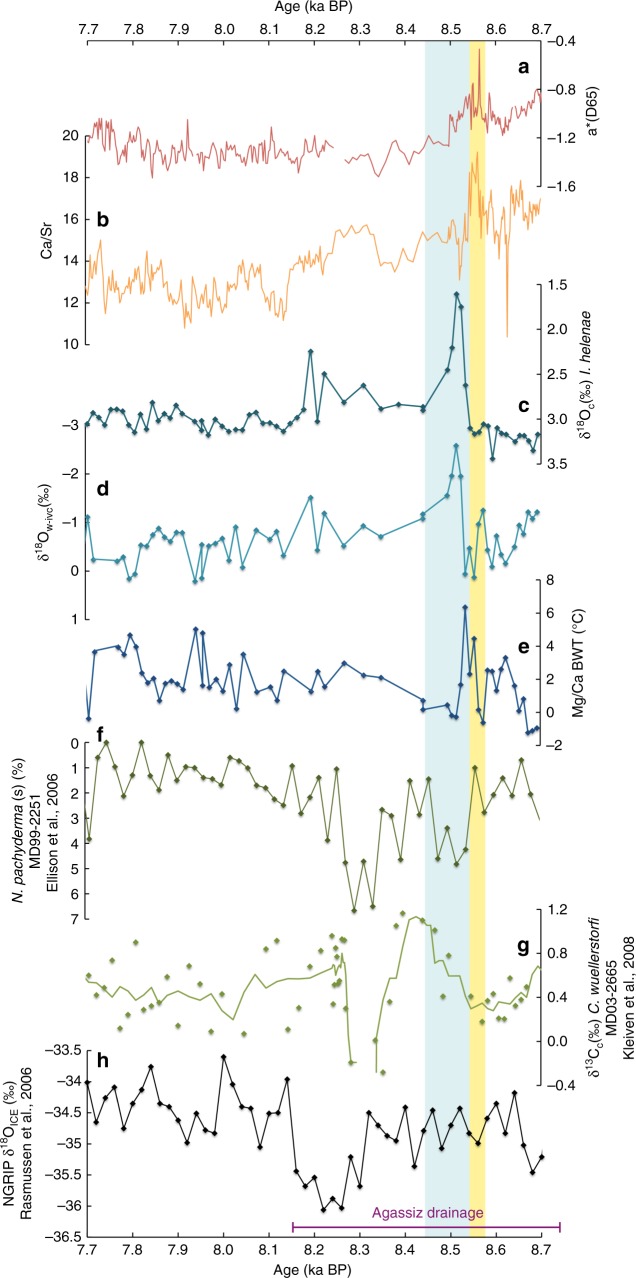
Effects of the freshwater pulse on North Atlantic circulation and temperature. Early Holocene high-resolution records of core MSM45-19-2 (**a**–**e**) are compared with Atlantic Meridional Overturning Circulation records of MD99-2251^7^ (**f**) and MD03-2665^8^ (**g**) as well as the atmospheric temperature record in NGRIP^4–6^ (**h**). Also shown is the age of the Agassiz drainage event with 1σ uncertainties (8.16–8.74 ka before present)^9^ denoted by a purple line. The yellow vertical bar marks the initial Lake Agassiz-Ojibway outburst, while the blue bar marks the Hudson Bay Ice Saddle collapse

The most significant freshening of subsurface waters is centered at 8.5 ka BP, with apparently two meltwater pulses associated with two cold spells between 8.6 and 8.5 ka BP (Fig. 4d, e). This interval is also characterized by increased levels of red sediment color (Fig. 4a). Based on the age model proposed here, it lies within the 1-sigma age constraints for the red-colored, hematite-rich sediment layer in Hudson Strait and Bay presented by Barber et al.^9^ (8.74–8.16 ka BP, purple line in Fig. 4). The dispersal of red-colored sediment downstream of northern central Hudson Bay, where red-colored erratics have been deposited by terrestrial ice flow centers during the glacial^11,44^, was ascribed to the subglacial drainage of LAO^9,12,13^. The first and less severe of the two freshwater pulses, at about 8.58 ka BP in the δ^18^O_w-ivc_ record, correlates with the highest peaks in red sediment color (a*D65) and detrital carbonate (Ca/Sr). Although Jennings et al.^38^ associate the red bed with the opening of the Tyrell Sea, we interpret the first freshening and input of red sediment and detrital carbonate to represent the initial phase of the subglacial LAO outburst flood (yellow bar in Fig. 4). A minor freshwater perturbation during the initial LAO drainage event is consistent with climate modeling studies that have shown the lake outburst itself not to be sufficient to induce the 8.2 ka cooling event in its full duration^29,31^. Importantly, the most prominent freshwater signal is recorded during a second pulse at 8.5 ka BP, following but not corresponding to the LAO-related peaks in detrital carbonate and sediment redness (blue bar in Fig. 4). This second and largest freshwater pulse was, therefore, not related to the initial lake outburst and sediment dispersal itself.

### Freshening caused by Hudson Bay Ice Saddle collapse

The δ^18^O_w-ivc_ reduction of about −2.5‰ between 8.55 and 8.45 ka BP (blue bar in Fig. 4) is the most pronounced benthic freshwater signal so far recorded in Holocene Labrador Sea sediments and implies an immense volume of freshwater to dilute the shelf waters down to 200 m depth for about 100 years. Remarkably, the benthic foraminifera revealing the major freshwater peak at 1043 cm core depth have a radiocarbon age about 300–600 years older than those dated just below and above, respectively (Supplementary Table 1). As there is no sedimentological evidence for any disturbance coeval with the ^14^C and δ^18^O excursions, we suggest that the old age was caused by glacial meltwater run-off. An older radiocarbon signature can be found in water masses from Hudson Bay and Strait as they are in contact with Paleozoic detrital carbonates that contain no more radiocarbon^12^. Additionally, freshwater that is primarily formed through subglacial or basal ice sheet melt can contain an extremely old radiocarbon imprint due to ancient carbon dioxide that can be stored in the ice for several thousand years^45^. While surface melting produces meltwater that is in equilibrium with atmospheric carbon dioxide, such as that stored in proglacial lakes, subsurface melting of glaciers prohibits meltwater contact and exchange with the atmosphere, which can lead to reservoir ages of up to 2700 years^46^. Hence, the old radiocarbon age of benthic foraminifera bathed by the second, much more prominent freshwater pulse is consistent with the drainage of old glacial meltwater rather than an outburst of an ice-dammed proglacial lake.

The timing and duration of the severe Labrador Shelf freshening seen in our record is consistent with the disappearance of the Hudson Bay Ice Saddle between 8.5 and 8.4 ka BP^32,33^. Based on our estimate of its areal extent (about 450,000 km^2^), using an updated ice sheet volume equation^34^, we calculate that the Hudson Bay Ice Saddle could have stored about 451,000 km^3^ of freshwater, a volume that may have been large enough to dilute Labrador Shelf waters down to 200 m depth. This scenario is also in agreement with an ice sheet model study that produced the largest early Holocene freshwater pulse during the separation of the Keewatin, Labrador, and Fox domes^29^. Thus, we propose that the severe Labrador Shelf freshening at 8.55–8.45 ka BP was caused by the melting and collapse of the Hudson Bay Ice Saddle, which, according to a General Circulation Model simulation experiment, would have been sufficient to trigger the 8.2 ka cooling event^31^.

The timing of the severe Labrador Shelf freshening indeed corresponds to the first of two surface cooling events apparent in deep-sea core MD99-2251 from the subpolar North Atlantic^7^ (Fig. 4f). However, only the second of the two surface temperature minima in core MD99-2251 correlates with the surface water cooling in core MD03-2665 south of Greenland^8^ (Fig. 4g) and the atmospheric temperature drop at 8.2 ka BP recorded in Greenland ice core NGRIP^4–6^ (Fig. 4h), which is also evident with a δ^18^O_c_ reduction of 2.3‰ at 8.2 ka in our record (Fig. 4c). Hence, the major Labrador Current freshening in our record predates the widespread atmospheric cooling event by about 200 years, a delay that appears too long compared to model studies that simulate climate responses to freshwater forcing within decades. However, freshwater hosing experiments by Renssen et al.^47^ simulated an AMOC disruption of about 145–320 years by using a Labrador Sea meltwater pulse of 467,000 km^3^ over 20–50 years, which is in the order of our volume estimate for the Hudson Bay Ice Saddle and the duration of our freshwater peak. An AMOC reduction of 145–320 years would be in harmony with the 200-year delay between the 8.2 event and the freshwater forcing seen in our record. Yet, whether the delay was caused by the response time of transferring the density perturbation to early Holocene North Atlantic deepwater convection sites or whether a major second freshwater injection was required to reach a full AMOC reduction at 8.2 ka BP is still debatable.

### Ice saddle collapse accelerated by subsurface warming

**Fig. 5:**
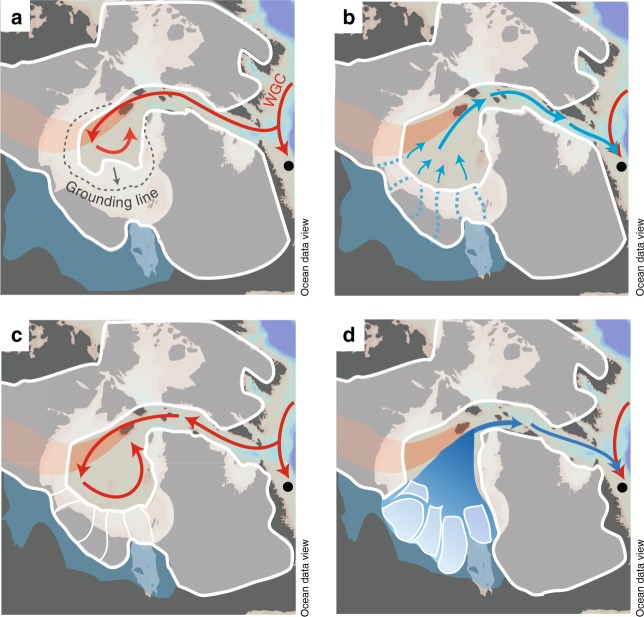
Schematic areal views of the Hudson Bay Ice Saddle collapse between 8.7–8.4 ka before present. A black circle marks the location of core MSM45-19-2 on the northern Labrador Shelf. **a** Inflowing warmer Atlantic waters from the West Greenland Current (red arrows) could have caused a retreat of the ice saddle grounding line in Hudson Bay. **b** As the Hudson Bay Ice Saddle became narrower (through subsurface warming) and lower (through surface ablation), it could no longer withstand the hydraulic pressure of Lake Agassiz-Ojibway with a lake level of 230 m above sea level^15^. The hydraulic pressure would have caused fractures and a partial uplift of the ice dam, which would have allowed the subglacial drainage of lake waters underneath the ice dam and through Hudson Strait into the Labrador Sea (blue arrows). The drainage would have stopped once the lake level dropped to sea level. **c** The fractured and now basally decoupled ice dam could have been further melted by inflowing warmer Atlantic waters. **d** The break-off of fault-bounded sections would have caused the collapse of the Hudson Bay Ice Saddle, leading to the discharge of large icebergs and high volumes of freshwater (blue arrows) into the Labrador Sea

The bottom water temperature record indicates two pulses of subsurface warming between 8.7 and 8.55 ka BP that are followed by cold freshwater pulses associated with the initial LAO outburst and the Hudson Bay Ice Saddle collapse (Fig. 4e). We relate the Labrador Shelf subsurface warming to the strengthening of the West Greenland Current after about 9.2 ka BP^48,49^ supplying warmer Atlantic waters to the western Labrador Sea with its westward retroflection (see Fig. 1). An early Holocene strengthening of the retroflected branch of the West Greenland Current is also evident in nearby core HU87033-017 from Cartwright Saddle^36^ that showed a gradual warming of surface waters prior to 9 ka BP. In parallel, the Hudson Strait Ice Stream, the largest ice stream of the LIS, retreated westward^32,33^ from about 10.2 ka BP, leaving Hudson Strait and central Hudson Bay ice-free by 8.2 ka BP, while major ice domes remained on land until 6.7 ka BP^34^. The coincidence of Labrador Shelf water warming and Hudson Strait Ice Stream retreat implies that inflowing warm waters may have accelerated the ice stream retreat. A similar process is described for the modern retreat of Jakobshavn Isbræ in western Greenland, where inflowing warm Irminger Current waters accelerate the ice stream flow and cause a retreat of the grounding line in the Jakobshavn ocean fjord^50^. A similar mechanism could have occurred in Hudson Strait, which can be considered as a fjord-like setting at that time (Fig. 5a). If the Hudson Strait Ice Stream flowed into a floating ice tongue or an embayed ice shelf, warmer ocean waters could have circulated beneath it, inducing basal melt and thinning, which would have reduced buttressing effects that provide a backpressure on ice stream flow^51^. The loss of buttressing would have increased velocities at the grounding line, which would have accelerated the retreat of the Hudson Strait Ice Stream. The opening of Hudson Strait and central Hudson Bay may have allowed more warm waters to circulate into central Hudson Bay underneath a thin layer of buoyant meltwater or an ice shelf. Here, it could have had a major impact on the saddle’s marine margin, causing a further retreat of the grounding line (Fig. 5a), in addition to surface ablation lowering the saddle’s altitude^29^. As the Hudson Bay Ice Saddle became narrower and lower, its reduced volume may not have been sufficient to withstand the water pressure caused by proglacial LAO with a surface elevation of 230 m above sea level^15^. This hydraulic pressure may have fractured the ice and could have caused a partial uplift of fault-bounded sections of the ice dam, allowing the subglacial drainage of LAO (Fig. 5b), similar to modern observations of the annual drainage of ice-dammed Hidden Creek Lake in Alaska^52^. The subglacial lake drainage is supported by the finding of erosive channels in the south-western Hudson Bay seabed^10,11^. The drainage would have stopped once the lake surface dropped to sea level height, leaving the lower part of the lake behind, while continuously inflowing warm subsurface waters may have further melted the ice dam that was fractured and decoupled from the bedrock (Fig. 5c). Moreover, the break-off of large icebergs from the heavily fractured ice would have eventually led to the collapse of the Hudson Bay Ice Saddle (Fig. 5d). Thus, we propose that inflowing warmer waters into Hudson Bay may have played an important role in the processes leading to the initial LAO drainage and the subsequent collapse of the Hudson Bay Ice Saddle.

Although, during the early Holocene, the LIS was in a highly negative mass balance mainly controlled by surface ablation^26,29,35^, our record implies that subsurface warming could have been an additional dynamical process that could have facilitated the instability of the ice dam and accelerated the collapse of the Hudson Bay Ice Saddle. Furthermore, our study presents the first high-resolution record of the sequence of events leading to the most severe early Holocene Labrador Sea freshening, suggesting that the lake drainage preceded the collapse of the Hudson Bay Ice Saddle. Owing to the timing of the outburst, the event could have had an amplifying effect on the Hudson Bay Ice Saddle collapse.

In summary, this study presents a high-resolution record from the northern Labrador Shelf that increases our understanding of the mechanisms involved in the final deglaciation of the LIS. We found direct geochemical evidence of a significant freshening at 8.5 ka BP that diluted Labrador Shelf waters down to 200 m depth. We associate this major subsurface/bottom water freshening with the Hudson Bay Ice Saddle collapse following the initial subglacial drainage of LAO, which in turn is marked by peaks in Ca/Sr and sediment redness at 8.55 ka BP. Our chronology suggests that the severe early Holocene Labrador Sea freshening could have initiated a major AMOC disruption associated with the 8.2 ka cold event. In addition, our record shows a subsurface warming in Labrador Shelf waters prior to the freshening, which we ascribe to a strengthening of the westward retroflection of the West Greenland Current. If these warmer Atlantic waters entered Hudson Bay, they could have played a significant role in accelerating the retreat of the Hudson Strait Ice Stream and in facilitating the Hudson Bay Ice Saddle collapse through subsurface warming. Thus, our study presents a scenario that can help to explain the rapidity of the ice saddle collapse and our findings may help to improve model simulations of the final LIS deglaciation by providing a mechanism additional to surface ablation.

## Methods

### Material

During the R/V *Maria S. Merian* cruise MSM45 in August 2015, a 1306 cm gravity core (MSM45-19-2) was recovered from the northernmost Labrador Shelf at 58°45.68 N, 61°56.25 W, at 202 m water depth (Fig. 1, Supplementary Fig. 1).

### Chronology

The chronology of core MSM45-19-2 is based on 21 AMS ^14^C measurements, which were carried out on mixed calcareous benthic foraminifera at the Leibniz Laboratory of Kiel University (CAU), Germany. Radiocarbon dates were calibrated to “calendar” scale using Calib 7.1^53^ based on the Marine13 dataset^54^ with reservoir corrections of Δ*R* = 144 ± 38 and 344 ± 38 years. Six AMS dates between 1043 and 1273 cm depth were excluded from linear interpolation (Supplementary Table 1).

### Light reflectance measurements

The light reflectance was measured in 1-cm intervals on the foil-covered, air-bubble-free, core surface with a hand-held Konica Minolta CM 600d spectrophotometer on board the research vessel. The spectrum of the reflected light was detected by a multi-segment light sensor at a 20 nm pitch between wavelengths of 400–700 nm. The a*(D65) value reflects the ratio of magenta (700 nm) and green (500 nm).

### X-ray fluorescence scanning

The bulk sediment elemental composition was analyzed at Kiel University, using an Avaatech XRF core scanner. The archive half of the core was carefully scraped to create a smooth surface before covering it with a 4-µm-thin SPEXCertiPrepUltralene foil to avoid contamination^55^. Each section was successively scanned with voltages of 10 kV (10 s, 750 µA, no filter), 30 kV (20 s, 500 µA, Pd-Thin filter), and 50 kV (40 s, 1750 µA, Cu filter) to measure the full suite of elements between Al and Ba in 1-cm resolution. Instrumental variance was monitored by measuring a set of standards (SARM4, JGa-1, JR-1, KGa-1) prior to each section and at the end of daily scanning.

### Mg/Ca measurements and temperature estimates

About 60 specimens of *I. helenae* were handpicked from the 200–315 µm fraction in 5-cm intervals downcore, weighed, and crushed between two glass plates. The crushed samples were then split for Mg/Ca and stable isotope measurements (see below). About two thirds of each crushed sample was transferred into pre-leached Eppendorf vials and cleaned following the full protocol of Martin and Lea^56^, including a reductive and oxidative cleaning step and a final leaching step with 0.001 N HNO_3_. After dissolving and diluting the samples in 0.1 N HNO_3_, they were measured with an inductively coupled plasma-optical emission spectrometry (ICP-OES) instrument with radial plasma observation at the Institute of Geosciences, Kiel University. The analytical error of Mg/Ca analyses was 0.1% relative standard deviation and accuracy was monitored with reference material JCP-1. Additional trace elements (Fe, Al, and Mn) were monitored to exclude possible contaminated or coated samples from the dataset. Based on 15 duplicate down-core sample measurements, we obtained a standard deviation of 0.07 mmol/mol Mg/Ca, which translates into 0.9 °C with respect to temperature estimates. For bottom water temperature reconstructions, we applied the calibration of Skirbekk et al.^57^. As the calibration is based on a temperature range of 1–4 °C, it may become less accurate when temperatures exceed 4 °C, which is the case for 25% of the samples reported here.

### Oxygen isotope measurements

About one third of the crushed samples of *I. helenae* were cleaned with ethanol absolute, decanted, and dried at 40 °C. Stable oxygen isotope analyses were carried out at the Leibniz Laboratory for Radiometric Dating and Stable Isotope Research in Kiel. A Finnigan MAT 253 mass spectrometer coupled with a Kiel IV carbonate preparation device was used and calibrated to the Vienna Pee Dee Belemnite (V-PDB) scale, which is applied to all δ^18^O_CaCO3_ analyses. Based on 15 duplicate down-core sample measurements, we obtained a standard deviation of 0.09‰. To estimate the δ^18^O_w_ (seawater), the value of calcitic δ^18^O_c_ (‰ V-PDB) was first translated into the V-SMOW scale by adding 0.27‰ and, together with the Mg/Ca-derived temperature, applied in the Shackleton equation *T* = 16.9 − 4.0 (δ^18^O_c_ − δ^18^O_w_)^58^. To correct for ice volume (δ^18^O_w-ivc_), a sea-level correction of 0.0083‰ per m (1‰ for 120 m of sea level) was derived from the relative sea level curve by Austermann et al.^59^. Based on duplicates, δ^18^O_w-ivc_ values have a standard deviation of 0.31‰. The bottom water signals (Mg/Ca BWT°C, δ^18^Ο_c_ and δ^18^Ο_w-ivc_) are based on infaunal benthic foraminifera *I. helenae*, which tend to live several centimeters deep in the sediment. This may explain the apparent lead of the bottom water signals compared to the sedimentological proxies Ca/Sr and a*(D65).

## Supplementary information

Supplementary Information

## Data Availability

The data sets generated during the study are available from the corresponding author A.A.L.
