# Supplementary material for: Labrador Sea freshening at 8.5 ka BP caused by Hudson Bay Ice Saddle collapse
Source: Nat Commun. 2019 Feb 4;10:586. doi: 10.1038/s41467-019-08408-6 (PMC6362222; doi:10.1038/s41467-019-08408-6)
Supplement: Supplementary file 1 — Supplementary Information [file 41467_2019_8408_MOESM1_ESM.docx]

**Supplementary Information**

Labrador Sea freshening at 8.5 ka BP caused by

Hudson Bay Ice Saddle collapse (Lochte et al.)

**Supplementary Information**

**
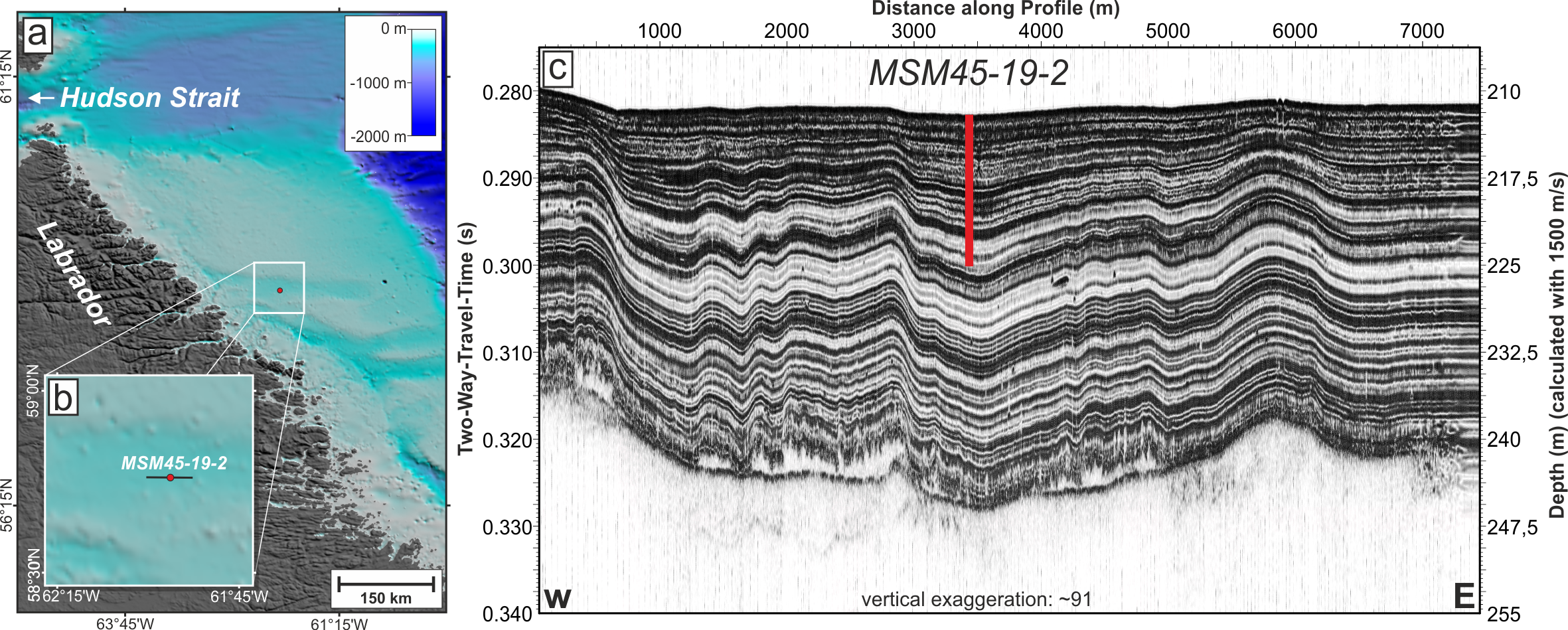
Supplementary Figure 1 |** Core location on the northern Labrador Shelf. a) Bathymetric map of the survey area with core location. b) Zoom-in to the core location of MSM45-19-2. c) Hull-mounted PARASOUND P70 echogram across the core location MSM45-19-2 displayed with a vertical exaggeration of ~91. The echogram shows well-stratified and undisturbed sedimentary units within the upper 40 ms (~30 m).

**Supplementary Table 1:** Twenty-one AMS radiocarbon dates obtained from mixed benthic foraminifera. Reported radiocarbon dates were calibrated in Calib7.1^1^ based on the Marine13 dataset^2^ with reservoir corrections (ΔR) of 144 ± 38 and 344 ± 38. The youngest sample KIA 51535 could not be calibrated with the Marine13 dataset. We assume an age younger than 1950 AD. Six AMS dates between 1043 and 1273 cm depth were excluded from linear interpolation due to anomalously old calendar ages (in italic).

| **AMS** | **Depth** | **Reported** | **Reservoir** | **1 sigma** | **2 sigma** | **Median** |
| --- | --- | --- | --- | --- | --- | --- |
| **laboratory** | **(cm)** | **^14^C age** | **correction** | **age range** | **age range** | **probability** |
| **number** |  | **(yr BP)** | **ΔR (^14^C yr)** | **(cal yr BP)** | **(cal yr BP)** | **age (cal yr BP)** |
| KIA 51535 | 3 | 204 ± 26 | 144 ± 38 |  |  | 0 |
| KIA 51536 | 103 | 2563 ± 25 | 144 ± 38 | 1989 – 2116 | 1915 – 2195 | 2055 |
| KIA 51537 | 203 | 4702 ± 35 | 144 ± 38 | 4697 – 4832 | 4583 – 4832 | 4761 |
| KIA 51538 | 303 | 5570 ± 34 | 144 ± 38 | 5740 – 5868 | 5665 – 5907 | 5799 |
| KIA 51539 | 403 | 6318 ± 37 | 144 ± 38 | 6536 – 6677 | 6468 – 6741 | 6610 |
| KIA 51540 | 503 | 6781 ± 36 | 144 ± 38 | 7118 – 7243 | 7010 – 7275 | 7169 |
| KIA 51541 | 603 | 6989 ± 38 | 144 ± 38 | 7312 – 7414 | 7254 – 7457 | 7362 |
| KIA 51542 | 703 | 7452 ± 39 | 344 ± 38 | 7539 – 7642 | 7474 – 7682 | 7584 |
| KIA 51543 | 803 | 7546 ± 42 | 344 ± 38 | 7601 – 7720 | 7561 – 7790 | 7666 |
| KIA 51544 | 903 | 7811 ± 40 | 344 ± 38 | 7864 – 7979 | 7795 – 8042 | 7926 |
| KIA 51545 | 1003 | 8097 ± 45 | 344 ± 38 | 8163 – 8295 | 8073 – 8351 | 8225 |
| KIA 52109 | 1013 | 8190 ± 40 | 344 ± 38 | 8256 – 8376 | 8179 – 8406 | 8311 |
| KIA 52110 | 1023 | 8270 ± 40 | 344 ± 38 | 8330 – 8439 | 8282 – 8522 | 8388 |
| KIA 52586 | 1033 | 8385 ± 40 | 344 ± 38 | 8424 – 8550 | 8377 – 8618 | 8495 |
| KIA 52587 | 1043 | 8990 ± 40 | 344 ± 38 | 9254 – 9398 | 9145 – 9443 | *9317* |
| KIA 52035 | 1048 | 8720 ± 40 | 344 ± 38 | 8880 – 9051 | 8753 – 9123 | *8964* |
| KIA 51546 | 1103 | 8805 ± 44 | 344 ± 38 | 8988 – 9146 | 8947 – 9260 | *9079* |
| KIA 51547 | 1203 | 8914 ± 50 | 344 ± 38 | 9112 – 9305 | 9030 – 9386 | *9213* |
| KIA 52588 | 1233 | 9105 ± 70 | 344 ± 38 | 9356 – 9511 | 9231 – 9572 | *9425* |
| KIA 52590 | 1273 | 8965 ± 40 | 344 ± 38 | 9220 – 9385 | 9116 – 9425 | *9287* |
| KIA 52591 | 1303 | 8765 ± 50 | 344 ± 38 | 8949 – 9121 | 8805 – 8818^a^ | 9029 |
|  |  |  |  |  | 8840 – 9240^b^ |  |
| Relative probabilities: a = 0.006; b = 0.994 | | | |  |  |  |

**Supplementary References**

1 Stuiver, M. & Reimer, P. J. Extended 14C Data Base and Revised CALIB 3.0 14C Age Calibration Program. *Radiocarbon* **35**, 215-230, doi:10.1017/S0033822200013904 (1993).

2 Reimer, P. J. *et al.* IntCal13 and Marine13 Radiocarbon Age Calibration Curves 0–50,000 Years cal BP. *Radiocarbon* **55**, 1869-1887, doi:10.2458/azu_js_rc.55.16947 (2013).
